# Inferior Displacement of the Mandibular Canal and Mental Foramen Enlargement in an Asymptomatic Intraosseous Schwannoma of the Mandible: A Case Report

**DOI:** 10.7759/cureus.113872

**Published:** 2026-08-03

**Authors:** Yoshiki Sato, Shintaro Yodogawa, Yurina Wakabayashi, Hiroki Nagayasu, Tsuyoshi Shimo

**Affiliations:** 1 Division of Reconstructive Surgery for Oral and Maxillofacial Region, Department of Human Biology and Pathophysiology, School of Dentistry, Health Sciences University of Hokkaido, Hokkaido, JPN; 2 Division of Oral Maxillofacial Surgery for Oral and Maxillofacial Region, Department of Human Biology and Pathophysiology, School of Dentistry, Health Sciences University of Hokkaido, Hokkaido, JPN

**Keywords:** inferior alveolar nerve, intraosseous schwannoma, mandibular canal remodeling, mandibular schwannoma, mental foramen, neurogenic bone remodeling, panoramic radiography, radiographic-clinical dissociation

## Abstract

Intraosseous schwannoma of the mandible is a rare benign nerve sheath tumor that often resembles an odontogenic lesion radiographically. As many reported cases are asymptomatic, the absence of neurosensory symptoms is not a specific diagnostic clue. We report a 33-year-old female with a mandibular lesion detected incidentally on panoramic radiography. She had no pain, swelling, or numbness, and neurosensory examination of the mental nerve distribution was normal. The involved teeth were vital and showed no mobility or percussion pain. Panoramic radiography revealed a well-defined unilocular radiolucency extending from the right mandibular premolar region to the mesial root of the first molar. Computed tomography showed a relatively homogeneous low-attenuation lesion, inferior displacement of the mandibular canal, smooth thinning and resorption of the buccal and lingual cortices, and remodeling and enlargement of the mental foramen, without root resorption. Thus, marked remodeling centered on the inferior alveolar nerve pathway was present despite preserved neurosensory and pulpal function, demonstrating radiographic-clinical dissociation. Surgical excision revealed an encapsulated yellowish-brown solid mass adherent to the inferior alveolar nerve near the mental foramen; funnel-shaped enlargement of the mental foramen was observed intraoperatively. Histopathological examination demonstrated mixed Antoni A and Antoni B areas, and the tumor cells were positive for S100 protein and neuron-specific enolase, confirming schwannoma. Mild postoperative hypoesthesia resolved within five months, and no recurrence was observed during the available two-year follow-up. The combination of mandibular canal displacement and mental foramen enlargement may suggest a neurogenic origin and aid differential diagnosis and nerve-preserving surgical planning, even in patients without neurosensory symptoms.

## Introduction

Schwannoma is a benign, usually encapsulated, slow-growing peripheral nerve-sheath tumor derived from Schwann cells [1,2]. Although schwannomas frequently occur in the head and neck region, oral lesions arise predominantly in soft tissues, including the tongue, floor of the mouth, palate, and buccal mucosa [2,3]. Intraosseous schwannoma of the jaws is exceptionally rare. A recent review of 102 reported intraosseous schwannomas across all skeletal sites found that 35 cases (34.3%) occurred in the head and neck, with the mandible representing the most frequently affected individual site (18 cases) [4]. A subsequent mandible-specific case series and literature update analyzed 99 patients, comprising 13 institutional cases and 86 previously reported cases [5]. In mandibular cases, the tumor may arise from or be closely associated with the inferior alveolar nerve or one of its branches [4-7].

Radiographically, intraosseous mandibular schwannoma usually appears as a well-defined unilocular or multilocular radiolucency but lacks pathognomonic radiographic characteristics [4-7]. This appearance overlaps substantially with that of odontogenic cysts, periapical lesions, ameloblastoma, and odontogenic keratocysts, making an accurate preoperative diagnosis difficult. Furthermore, both symptomatic and asymptomatic cases have been reported; therefore, the absence of pain, paresthesia, or numbness does not exclude a neurogenic tumor [4-6]. Accordingly, incidental detection and preserved neurosensory function alone provide limited diagnostic discrimination. As the mandibular canal and mental foramen correspond to the intraosseous course and exit of the inferior alveolar nerve, remodeling centered on these structures may provide a more anatomically specific clue to a neurogenic origin than the radiolucent appearance itself. In particular, mandibular canal widening or displacement and enlargement or remodeling of the mental foramen may indicate that the lesion arises from, or is closely associated with, the inferior alveolar nerve. These findings have been described in previous reports and reviews [4,5,8]. Although they are not pathognomonic, their presence should raise suspicion of a neurogenic lesion and may also alert clinicians to a close lesion-nerve relationship before surgery.

We report an intraosseous schwannoma of the mandible in a 33-year-old female who had no preoperative neurosensory disturbance and whose involved teeth remained vital. Computed tomography demonstrated inferior displacement of the mandibular canal, smooth thinning and resorption of the buccal and lingual cortices, and remodeling and enlargement of the mental foramen, without root resorption. Thus, clear remodeling centered on the mandibular canal and mental foramen was present despite preserved neurosensory and pulpal function, creating radiographic-clinical dissociation. This report emphasizes these nerve-related bone-remodeling features as potential imaging clues for including schwannoma in the differential diagnosis and for planning surgery with attention to preservation of the inferior alveolar and mental nerves.

## Case presentation

Patient information

A 33-year-old female (height, 159 cm; weight, 46 kg) was referred to our department in mid-May 2011 for evaluation and treatment of an intraosseous lesion in the right mandible.

Medical and family history

Her medical history was significant for rheumatoid arthritis. Detailed medication information, including the use of corticosteroids or immunosuppressive agents, was not available in the archived clinical records. A history of trauma to the mandible or prior surgery in the affected region was also not documented. Her family history was unremarkable.

History of present illness

In early May 2011, panoramic radiography obtained during dental treatment at a local dental clinic incidentally revealed a well-defined radiolucent lesion in the right mandibular premolar-to-molar region. The radiograph was not prompted by symptoms attributable to the lesion, as the patient had no pain, swelling, or neurosensory disturbance. She was subsequently referred to our department for further evaluation and treatment.

Clinical findings

At the initial visit, her face was symmetric, and neurosensory examination of the mental nerve distribution was normal. A specific examination of the regional lymph nodes was not documented in the available archived clinical records. Intraorally, the mucosa overlying the lesion showed no redness, swelling, ulceration, or abnormal discoloration. No obvious bony expansion or parchment-like sensation was detected from the right mandibular premolar region to the molar region. The right mandibular first and second premolars showed no mobility or percussion pain and responded positively to electric pulp testing.

Imaging findings

Panoramic radiography showed a well-defined unilocular radiolucency extending from the right mandibular premolar region to the mesial root of the first molar. The lesion encompassed the mental foramen and measured 17 x 28 mm. It contacted the roots of the right mandibular first premolar, second premolar, and first molar, but no obvious root resorption or displacement was observed.

**Figure 1:**
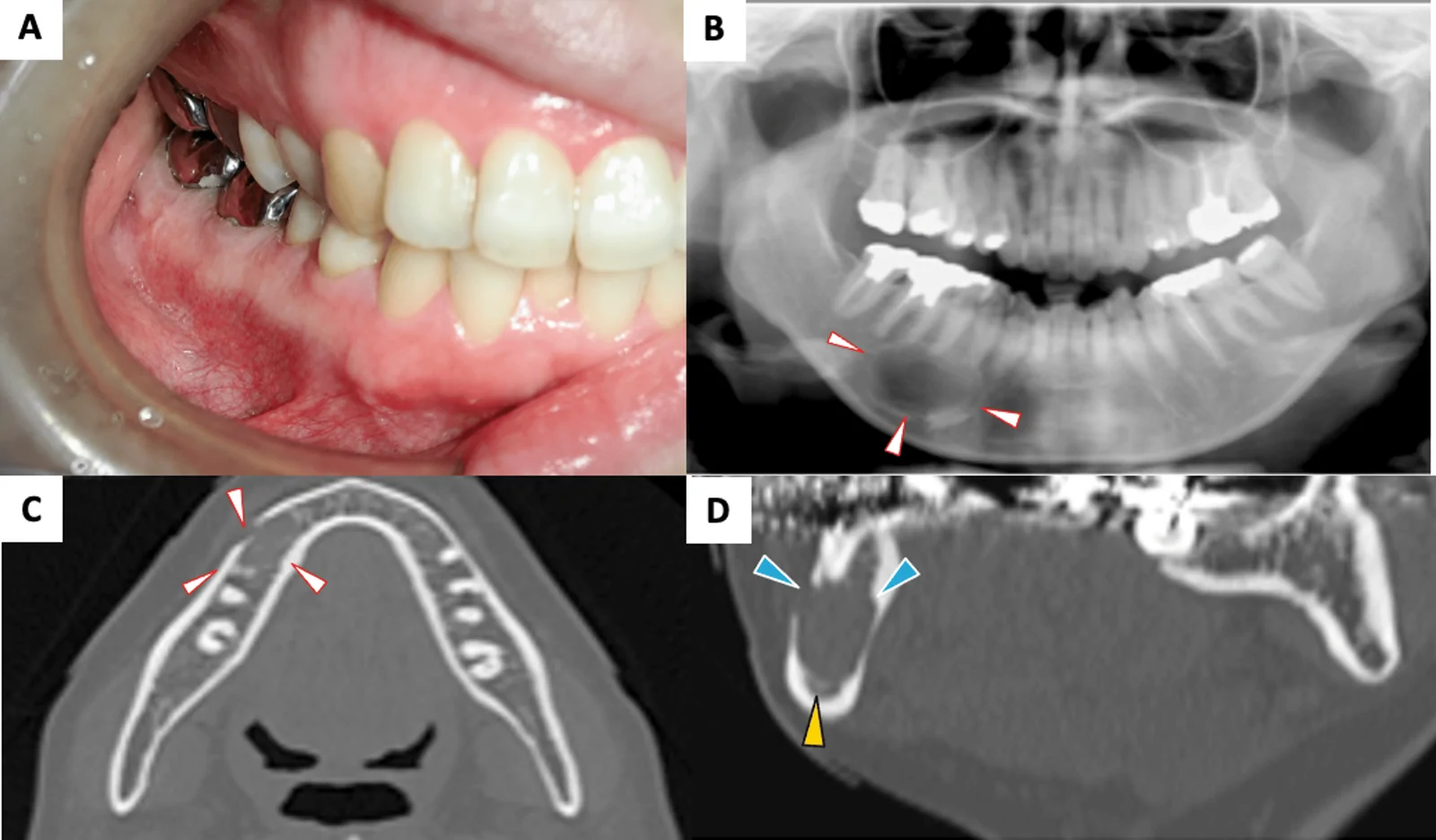
Clinical and radiographic findings at the initial visit. (A) Intraoral photograph showing no obvious swelling, mucosal discoloration, or ulceration in the right mandibular premolar-to-molar region. (B) Panoramic radiograph showing a well-defined unilocular radiolucency extending from the right mandibular premolar region to the mesial root of the first molar (red arrowheads), without obvious root resorption. (C) Axial computed tomography image showing smooth thinning and resorption of the buccal and lingual cortices (red arrowheads). (D) Reformatted computed tomography image showing enlargement of the mental foramen (blue arrowheads) and inferior displacement of the mandibular canal (yellow arrowhead).

Computed tomography showed a well-defined lesion with a relatively homogeneous low-attenuation internal structure and no evident internal septa. Quantitative Hounsfield unit measurements were not available from the archived imaging records. The lesion displaced the mandibular canal inferiorly and caused smooth thinning and resorption of both the buccal and lingual cortical plates. Cortical remodeling around the mental foramen resulted in its enlargement. The root apices of the right mandibular first and second premolars protruded into the lesion. Collectively, these findings represented bone remodeling centered on the mandibular canal and mental foramen (Figure 1).

Clinical diagnosis

The clinical diagnosis was a benign intraosseous lesion of the right mandible. The differential diagnosis included an odontogenic cyst, a benign odontogenic tumor, a simple bone cyst, a vascular lesion, and a neurogenic tumor. A simple bone cyst was considered because of the well-defined unilocular radiolucent appearance; however, it could not be definitively excluded on imaging alone. A periapical inflammatory lesion was considered less likely because the adjacent teeth were vital and showed no mobility or percussion pain. Although the well-defined unilocular appearance suggested a benign lesion, the radiographic findings alone did not permit definitive exclusion of cystic, vascular, or other intraosseous lesions. However, the inferior displacement of the mandibular canal and remodeling and enlargement of the mental foramen raised suspicion of a lesion arising from or closely associated with the inferior alveolar nerve.

Treatment and clinical course

Surgical excision was performed under general anesthesia in May 2011. Although the right mandibular first and second premolars showed positive pulp vitality, preoperative root canal treatment was performed because their root apices protruded into the lesion and were expected to be exposed during tumor removal. This treatment permitted subsequent root-end management, including apicoectomy of the exposed apices, while preserving the teeth.

During surgery, an incision was made along the cervical region from the right mandibular canine to the first molar, and a mucoperiosteal flap was elevated. Intraoperatively, the mental foramen was enlarged and showed a funnel-shaped configuration caused by bone resorption inferior to the root apex of the right mandibular first premolar.

**Figure 2:**
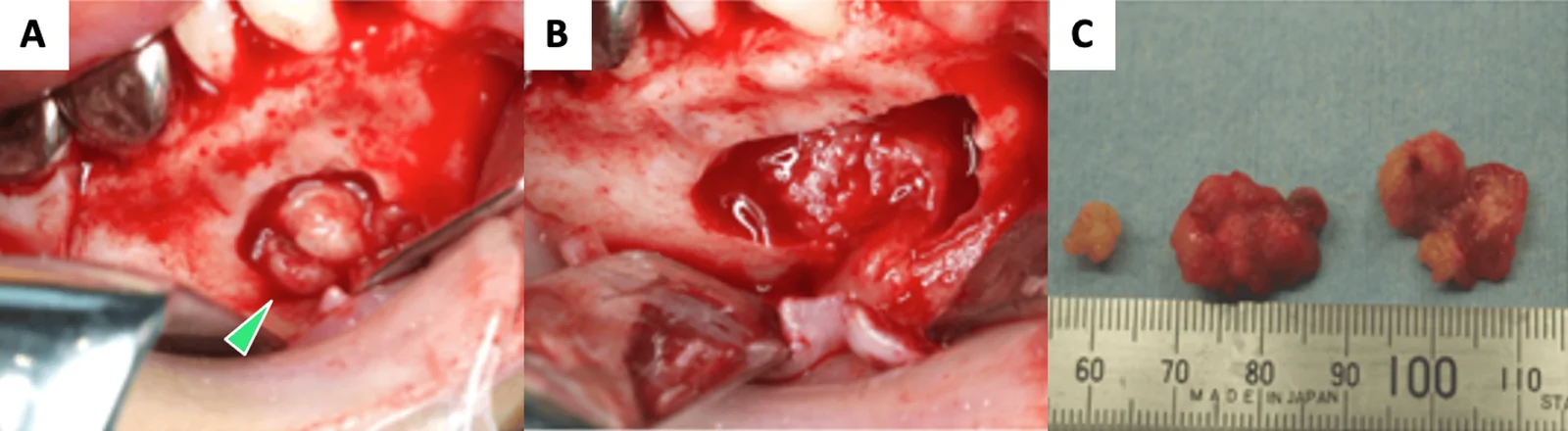
Intraoperative and macroscopic findings. (A) Intraoperative photograph showing the tumor exposed at the enlarged mental foramen. The green arrowhead indicates the funnel-shaped bony enlargement observed intraoperatively. (B) Intraoperative photograph showing the bone cavity after tumor removal. (C) Photograph of the excised specimen showing yellowish-brown solid tumor fragments.

The buccal cortical bone around the mental foramen was removed to expose the lesion. The lesion was an encapsulated, yellowish-brown solid mass with a firm, elastic consistency. The presence of an encapsulated solid mass, rather than an empty or fluid-containing bone cavity, excluded a simple bone cyst intraoperatively. The tumor was minimally adherent to the surrounding bone and was relatively easy to separate, except near the mental foramen, where it was firmly adherent to the inferior alveolar nerve. This direct anatomical relationship increased suspicion of a neurogenic tumor; however, the definitive diagnosis was established by histopathological and immunohistochemical examination (Figure 2).

After removal of the lesion, apicoectomy was performed on the exposed roots of the right mandibular first premolar, second premolar, and mesial root of the first molar. The bone cavity was smooth after tumor removal, and its inner surface was further trimmed with a bone bur. Mild hypoesthesia in the mental nerve distribution developed postoperatively. Details of the postoperative medications, including whether any medication was prescribed specifically for the hypoesthesia, could not be confirmed from the available archived clinical records. Sensation recovered within five months without any documented persistent neurosensory deficit. Clinical follow-up data were available for two years after surgery, during which no recurrence was observed. Information regarding the patient’s clinical course beyond this period was not available in the archived records.

Pathological findings

**Figure 3:**
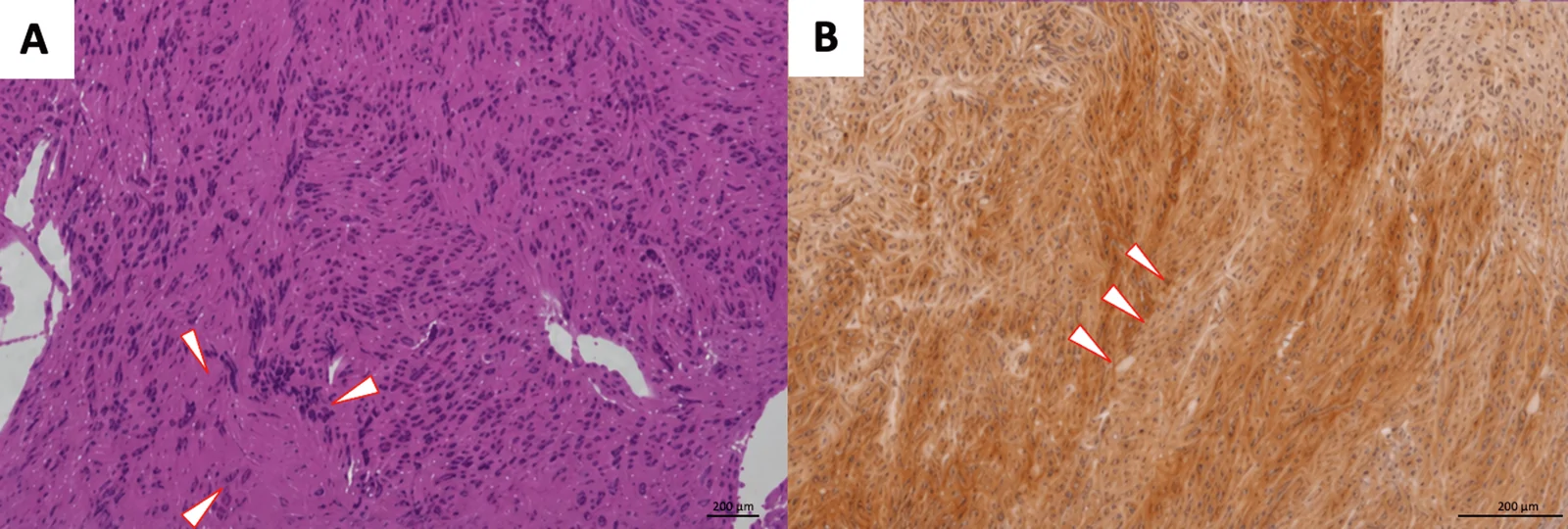
Histopathological findings and S100 immunohistochemistry. (A) Hematoxylin and eosin staining showing spindle-cell proliferation with an Antoni A-like fascicular arrangement and focal nuclear palisading (red arrowheads). (B) S100 immunohistochemical staining showing diffuse positivity in the spindle tumor cells (red arrowheads). Scale bars: 200 μm.

Histopathological examination showed a schwannoma composed of mixed Antoni A and Antoni B areas. Hematoxylin and eosin staining demonstrated tumor cells with spindle-shaped to oval nuclei densely arranged in fascicular or whorled patterns, with focal nuclear palisading corresponding to Antoni A areas. Other areas showed loosely arranged tumor cells in a reticular pattern, corresponding to Antoni B areas. Immunohistochemically, the tumor cells were positive for S100 protein and neuron-specific enolase, supporting the diagnosis of schwannoma. SOX10 immunohistochemistry was not included in the original diagnostic panel. Nevertheless, the diagnosis was established based on the characteristic histomorphological features, including mixed Antoni A and Antoni B areas and focal nuclear palisading, together with the immunohistochemical findings (Figure 3).

## Discussion

Intraosseous schwannoma of the mandible remains diagnostically challenging because its conventional radiographic appearance overlaps with that of more common odontogenic lesions. A systematic review, an imaging review, and the recent large series by Li et al. showed that these tumors most often present as well-defined unilocular or multilocular radiolucencies, whereas their clinical manifestations range from incidental findings to swelling, pain, and neurosensory disturbance [4-6]. Similarly, the cases summarized in Table 1 were frequently considered preoperatively to represent radicular or residual cysts, other odontogenic cysts, or ameloblastoma [9-14]. A well-defined radiolucency alone therefore provides little evidence for schwannoma.

**Table 1:** Recent case reports and the present case of intraosseous mandibular schwannoma with odontogenic lesion-like radiographic findings or inferior alveolar nerve involvement, with attention to mandibular canal and mental foramen remodeling. Abbreviations: NR, not reported; y, years; mo, months; preop, preoperative; postop, postoperative.

Author (year)	Age	Sex	Symptoms	Radiographic findings	Preoperative diagnosis	Treatment	Outcome
Iwai et al. (2022) [9]	45	F	Occlusal pain; no preoperative paresthesia	Well-defined cystic lesion around the mandibular first molar roots	Radicular cyst	Extraction and tumor removal	No recurrence at 7.5 y; postoperative paresthesia persisted
Ramesh et al. (2022) [10]	33	M	Incidentally detected	Small well-defined mandibular radiolucency	NR	NR	NR
Kharbouch et al. (2022) [11]	57	NR	Lower lip paresthesia; painless swelling	Well-circumscribed radiolucency with mandibular canal enlargement	NR	Enucleation with nerve preservation	Sensory recovery at 6 mo; no recurrence at 2 y
Mehta et al. (2024) [12]	NR	F	Facial swelling	Multilocular radiolucency of the posterior mandible with root resorption	NR	NR	NR
Alabdulkarim et al. (2024) [13]	40	F	Mandibular swelling	Unilocular radiolucency with cortical thinning/breakthrough	Schwannoma by biopsy	Resection, extraction, bone grafting, and reconstruction plate fixation	No recurrence or paresthesia at 1 y
Vijayalakshmi et al. (2024) [14]	38	F	Swelling; occasional tingling	Unilocular radiolucency near the mental foramen with cortical breach	Residual cyst	Biopsy, enucleation, and miniplate fixation	No recurrence at 3 mo; tingling persisted
Present case	33	F	Asymptomatic; no neurosensory disturbance	Unilocular radiolucency with mandibular canal displacement and mental foramen enlargement	Benign intraosseous lesion of the mandible	Surgical excision with endodontic treatment and apicoectomy	Hypoesthesia resolved at 5 mo; no recurrence at 2 y

The diagnostically relevant feature in the present case was not the radiolucency itself but its relationship with the inferior alveolar nerve pathway. Computed tomography demonstrated inferior displacement of the mandibular canal, smooth thinning and resorption of both the buccal and lingual cortices, and remodeling and enlargement of the mental foramen, without root resorption. Mandibular canal enlargement or displacement and changes around the mental foramen have also been described in previous reports and reviews [4,5,8,11,14]. Collectively, these changes are consistent with nerve-related bone remodeling because they are centered on the mandibular canal and its exit at the mental foramen. They are neither pathognomonic nor a diagnostic triad; however, their presence in a well-defined mandibular radiolucency should increase suspicion of a lesion arising from or closely associated with the inferior alveolar nerve. Intraoperatively, the enlarged mental foramen showed a funnel-shaped configuration, and the tumor was adherent to the inferior alveolar nerve near the foramen. These operative findings supported the anatomical relationship suggested by computed tomography; however, the funnel-shaped appearance was an intraoperative observation specific to this case.

Another important feature was the radiographic-clinical dissociation. Despite clear remodeling of the mandibular canal and mental foramen, the patient had no pain, numbness, or other neurosensory disturbance in the mental nerve distribution. The adjacent teeth showed no mobility or percussion pain and retained pulp vitality. Previous reports have included both symptomatic and asymptomatic patients, indicating that the absence of neurosensory symptoms cannot exclude an intraosseous schwannoma [5,6,9-14]. The combination of marked bone remodeling and preserved nerve function may reflect the slow, encapsulated growth of schwannoma, which can gradually displace its bony surroundings without causing early functional impairment. Although inferential, this interpretation may explain why radiographic alteration of the nerve pathway can precede clinically detectable sensory loss. Consequently, evaluation of the mandibular canal and mental foramen should not depend on the presence of paresthesia or numbness.

Three-dimensional imaging is important when a radiolucent lesion is located adjacent to the mandibular canal or mental foramen. Panoramic radiography demonstrates the overall lesion but may not adequately show buccolingual cortical changes or the precise relationship with the neurovascular bundle. Computed tomography or cone-beam computed tomography can define cortical remodeling and canal displacement, whereas magnetic resonance imaging may further clarify continuity with the nerve and internal soft-tissue characteristics [15,16]. Recognition of possible nerve-related bone remodeling before surgery may therefore influence the differential diagnosis, the selection of additional imaging, and counseling regarding the risk of postoperative sensory disturbance.

Histopathological examination remains essential for definitive diagnosis. The present tumor contained mixed Antoni A and Antoni B areas, with fascicular and whorled proliferation of spindle cells, focal nuclear palisading, and more loosely arranged reticular areas. Diffuse immunoreactivity for S100 protein and neuron-specific enolase supported Schwann cell differentiation [1,17,18]. These findings, together with the encapsulated gross appearance and attachment to the inferior alveolar nerve, established the diagnosis of schwannoma.

Complete surgical excision is generally associated with a favorable prognosis, but preservation of nerve function is an important consideration when the lesion is adherent to the inferior alveolar or mental nerve [5,6,19]. A systematic review of 93 gnathic intraosseous schwannomas reported a recurrence rate of 5.4%, with a mean follow-up period of 22.9 months [6]. In a more recent institutional series of 13 patients with intraosseous schwannoma of the mandible, no recurrence was observed during follow-up [5]. Although recurrence appears uncommon, the limited and heterogeneous follow-up periods in published reports support continued long-term postoperative surveillance. In the present case, most of the encapsulated tumor was readily separated from the surrounding bone, whereas dissection near the mental foramen was difficult because of attachment to the inferior alveolar nerve. Mild postoperative hypoesthesia resolved within five months, and no recurrence was observed during the available two-year follow-up. Recognition of the lesion-nerve relationship before surgery may assist surgical planning and enable appropriate preoperative counseling regarding postoperative neurosensory disturbance.

This report has several limitations. It describes a single retrospective case and therefore cannot establish the sensitivity or specificity of the proposed imaging features. The available computed tomography data did not permit objective morphometric assessment of the mental foramen, and the funnel-shaped appearance was based on an intraoperative observation. Preoperative magnetic resonance imaging was not performed. In addition, SOX10 immunohistochemistry was not included in the original diagnostic panel, although the diagnosis was supported by characteristic histomorphological findings and immunoreactivity for S100 protein and neuron-specific enolase. Clinical follow-up data were available for only two years; therefore, longer-term recurrence and neurosensory outcomes could not be assessed. Moreover, comparisons with previous cases were based largely on heterogeneous case reports with incomplete imaging descriptions. Nevertheless, inferior displacement of the mandibular canal and enlargement of the mental foramen, particularly when accompanied by preserved neurosensory and pulpal function, may suggest a neurogenic origin. These findings should prompt careful assessment of the lesion-nerve relationship and consideration of nerve-preserving surgical planning, while histopathological examination remains necessary for definitive diagnosis.

## Conclusions

We report an intraosseous schwannoma of the mandible showing inferior displacement of the mandibular canal, smooth cortical thinning and resorption, and enlargement of the mental foramen despite preserved neurosensory and pulpal function. This radiographic-clinical dissociation demonstrates that marked remodeling of the inferior alveolar nerve pathway can occur without preoperative sensory symptoms. Although these findings are not pathognomonic, their combination may support consideration of a neurogenic tumor in the differential diagnosis of a well-defined radiolucent lesion adjacent to the mandibular canal or mental foramen. Careful three-dimensional assessment of the lesion-nerve relationship may aid nerve-preserving surgical planning and counseling regarding postoperative neurosensory disturbance. Histopathological examination remains essential for definitive diagnosis. Because this report is based on a single case, the diagnostic value of these imaging features requires validation in additional cases and larger case series before broader clinical recommendations can be established.
